# Effects of Ginger Extract on Laying Performance, Egg Quality, and Antioxidant Status of Laying Hens

**DOI:** 10.3390/ani9110857

**Published:** 2019-10-23

**Authors:** Chao Wen, Yunfeng Gu, Zhengguo Tao, Zongjia Cheng, Tian Wang, Yanmin Zhou

**Affiliations:** 1 College of Animal Science and Technology, Nanjing Agricultural University, Nanjing 210095, China; 2 Leader Bio-technology Co., Ltd., Guangzhou 510663, China

**Keywords:** antioxidant status, egg quality, ginger extract, laying hen

## Abstract

**Simple Summary:**

The application of in-feed antibiotic growth promoters was banned in many countries due to their negative effects, and several kinds of feed additives were widely investigated as antibiotic alternatives, in which natural plant-derived products received much attention due to their environmentally friendly properties and numerous biological activities. Ginger (*Zingiber officinale* Roscoe), a widely used herbal medicine and spice, was proven to have potential as an antibiotic alternative in poultry feed, but there is little literature on the efficacy of ginger extract (GE), which has concentrated bioactive compounds with high bioavailability. Our results showed that dietary GE supplementation increased egg weight, improved egg quality, and reduced the yolk cholesterol content of laying hens. Decreased serum activities of alanine transaminase and aspartate transaminase and improved antioxidant status were observed in the GE group. Our study demonstrated the potential benefits of GE in laying hens.

**Abstract:**

The objective of this study was to investigate the effects of ginger extract (GE) as a dietary supplement for laying hens. A total of 40-week-old 288 Hyline Brown laying hens were randomly divided into two groups with six replicates, and fed a basal diet with or without 100 g/t GE for eight weeks. Dietary GE supplementation increased egg weight, albumin height, and Haugh unit of eggs, and decreased yolk cholesterol content and activities of alanine transaminase and aspartate transaminase in serum at eight weeks. Moreover, GE resulted in higher total superoxide dismutase (T-SOD) activity and lower malondialdehyde (MDA) content in yolk at four and eight weeks and in serum. It was concluded that GE was effective in increasing egg weight and improving the egg quality and antioxidant status of laying hens.

## 1. Introduction

With the increase in concerns about environmental and food safety, the application of antibiotic growth promoters in laying hens was banned in many countries, and numerous studies were conducted to investigate the potential of many additives as antibiotic alternatives in recent years, in which natural plant-derived products received much attention due to their environmentally friendly properties and numerous biological activities [1,2]. For example, the positive effects of several plant-derived products on egg production, egg quality, and antioxidant capacity of laying hens were demonstrated in recent studies [3,4,5]. As one of most common natural nutraceuticals, which refer to food-derived products with potential pharmaceutical activity [6,7,8], ginger (*Zingiber officinale* Roscoe) was used worldwide as herbal medicine and spice for many years due to its medical and culinary characteristics. It is effective for the treatment of arthritis, fever, vomiting, migraine, hypercholesterolemia, and ulcer [9]. The most important bioactive components of ginger responsible for its pungent taste and pharmacological activities were shown to be gingerols, including 6-, 8-, 10-gingerol, etc. [10]. Gingerols are a group of phenolic compounds present as yellow oil at room temperature, and they exhibit a host of biological activities, ranging from anticancer to antioxidant, antimicrobial, anti-inflammatory, and antiallergic activities, as well as various central nervous system activities [11]. Gingerols were proven to alleviate oxidative stress of animals induced by mycotoxins, heavy metals, aging, etc. [12,13,14].

The potential benefits of ginger application as an antibiotic alternative in poultry production were demonstrated [15]. Several studies showed the positive effects of ginger on egg production, egg quality, and the antioxidant status of laying hens [16,17,18]. As ginger contains low concentrations of gingerols that are enclosed in the cell wall and not easily absorbed by animals, ginger extract (GE) with concentrated gingerols may have better bioavailability and is, thus, more convenient to be used as a feed additive. However, based on our knowledge, there was little research conducted to evaluate the efficacy of GE in laying hens. Therefore, the objective of this study was to investigate the effects of GE on laying performance, egg quality, and the antioxidant status of serum and yolk in laying hens.

## 2. Materials and Methods

### 2.1. Experimental Design, Diets, and Husbandry

All procedures were approved by Nanjing Agricultural University Institutional Animal Care and Use Committee (Certification No.: SYXK (Su)2017-0007).

The GE used in this study was provided by Leader Bio-technology Co., Ltd. (Zhuhai, Guangdong, China) and prepared as follows: briefly, fresh ginger roots were dried, ground, and extracted by subcritical butane extraction. Then, butane was removed by reduced pressure, and extracted ginger oil was coated with starch and gelatin to obtain GE in powder form. The content of total gingerols in GE was 40.3 g/kg, which was composed of 78.3% 6-gingerol, 10.2% 8-gingerol, and 11.5% 10-gingerol as analyzed by high-performance liquid chromatography. Briefly, GE was dissolved in distilled water assisted by ultrasound. Then, hexane was added and mixed, followed by centrifugation at 3000 r/min for 5 min in order to separate the hexane phase. The amount of gingerols in hexane was quantified as previously described [19]. Gingerols were chromatographically separated by a sub-2-µm particle column. Acetonitrile–water was used as the mobile phase at a flow rate of 1.0 mL/min, and the detection wavelength was set at 280 nm.

**Figure 1:**
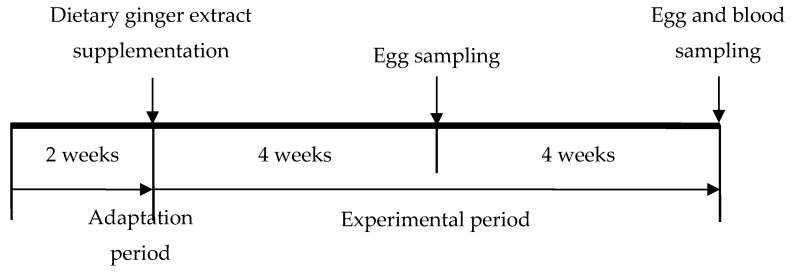
Scheme of experimental design.

A total of 288 Hyline Brown laying hens (40 weeks of age) were used in this study. After two weeks of adaptation period, the hens were allocated to two groups with six replicates of 24 hens (Figure 1). A commercial corn–soybean meal diet (Table 1) with or without GE (100 g/t) was used for an eight-week study. Hens were allowed free access to mash feed and water throughout the experiment and were exposed to a a 16-h/8-h light/dark cycle. Egg production and egg weight were recorded daily and feed consumption was recorded weekly per replicate. Egg mass and feed-to-egg ratios were calculated.

### 2.2. Sample Collection

At four and eight weeks of the experimental period, one egg per replicate was randomly selected for an egg quality assay, and the yolk was frozen at −20 °C until analysis. At eight weeks, one hen was randomly selected, and blood samples were taken from the wing vein, centrifuged at 3000× *g* for 15 min at 4 °C to separate the serum, which was frozen at −20 °C for further analysis.

### 2.3. Egg Quality Assay

Eggshell strength was measured by a compression tester (Model-II, Robotmation, Tokyo, Japan), and shell thickness was the average value of measurements at three points (blunt end, equator, and sharp end) by a micrometer. Yolk color, albumen height, and Haugh unit were analyzed by an egg multi-tester (EMT-7300, Robotmation, Tokyo, Japan). The egg yolk, albumin, and shell were weighed to calculate their percentages of egg weight.

### 2.4. Yolk Fat and Cholesterol Contents

Yolk fat content was determined by the Folch method [20]. Briefly, 0.4 g of yolk was homogenized with 6 mL of a chloroform/methanol (2/1) mixture at room temperature. Then, 2 mL of water was added, and the mixture was agitated and centrifuged at 2500 r/min for 10 min. The upper phase was removed by siphoning, and the lower phase containing lipids was filtered to a pre-weighed tube. The filtrate was then evaporated under a nitrogen stream and weighed again to calculate yolk fat content. Cholesterol content was determined as previously described [21]. Briefly, 0.1 g of yolk was mixed thoroughly with 0.3 mL of 33% (*w*/*v*) KOH and 3 mL of 95% ethanol, and then placed in a 60 °C water bath for 15 min. After cooling, 10 mL of hexane and 3 mL of distilled water were added and mixed. Appropriate aliquots of hexane layer were pipetted into a colorimeter tube, and the solvent was evaporated under nitrogen. Then, 2 mL of *o*-phthalaldehyde and 1 mL of concentrated sulfuric acid were added and mixed, and absorbance was read at 550 nm using a spectrophotometer.

### 2.5. Serum Transaminase Activities

Serum alanine transaminase (ALT) and aspartate transaminase (AST) activities were determined by commercial kits (Jiancheng Bioengineering Institute, Nanjing, China).

### 2.6. Antioxidant Status

Total superoxide dismutase (T-SOD) activity and malondialdehyde (MDA) content in yolk and serum were measured to evaluate the antioxidant status of laying hens. After thawing, the yolk was homogenized (1:9, *w*/*v*) by ice-cold physiological saline solution (for T-SOD assay) or anhydrous ethanol (for MDA assay), and then centrifuged at 5000× *g* for 10 min at 4 °C to collect the supernatant. The T-SOD activity and MDA content in the supernatant were measured using the nitrite method [22] and thiobarbituric acid method [23], respectively, using commercial kits (Jiancheng Bioengineering Institute, Nanjing, China).

### 2.7. Statistical Analysis

All data were analyzed as a completely randomized design using one-way ANOVA (SPSS, 2008). The differences were considered to be significant at *p* < 0.05. The ANOVA test with *p*-values between 0.05 and 0.10 was considered as a trend toward significance.

## 3. Results

### 3.1. Laying Performance

Compared with the control group, dietary GE supplementation increased (*p* < 0.05) egg weight and tended (*p* < 0.1) to increase egg mass (Table 2). The average daily feed intake was not affected significantly, although an increasing trend (*p* < 0.1) was also observed in GE group. There was no difference in laying rate or feed to egg ratio.

### 3.2. Egg Quality

The laying hens fed diets supplemented with GE had higher (*p* < 0.05) albumin height and Haugh unit of eggs than the control hens at both four and eight weeks, but yolk color, eggshell strength, and eggshell thickness did not differ (Table 3). The percentages of eggshell, yolk, and albumin were similar between the groups.

### 3.3. Yolk Fat and Cholesterol Contents

The contents of fat and cholesterol in yolk did not differ between the groups at four weeks (Table 4). However, laying hens fed diets supplemented with GE had lower (*p* < 0.05) yolk cholesterol content than the control group at eight weeks, but yolk fat content was still not affected.

### 3.4. Serum Transaminase Activities

The activities of ALT and AST in serum were reduced (*p* < 0.05) by dietary GE supplementation compared with the control group (Table 5).

### 3.5. Antioxidant Status

Laying hens fed diets supplemented with GE had higher (*p* < 0.05) T-SOD activity and lower (*p* < 0.05) MDA content in yolk at four and eight weeks and in serum than the control hens (Table 6).

## 4. Discussion

This study indicated that dietary GE supplementation increased egg weight and tended to increase egg mass. There was no obvious difference in average daily feed intake, although an increasing trend was observed in the GE group, suggesting that GE might increase egg weight by increasing feed utilization but not feed intake. Our finding was similar to the results of Zhao et al. [24], who observed higher egg mass in laying hens fed diets supplemented with ginger powder. Ademola et al. [25] also reported that mixtures of ginger and garlic significantly increased the egg weight of laying hens. However, Yang et al. [26] reported that ginger root supplementation improved laying rate and feed conversion ratio but did not affect egg weight or egg mass. The discrepancy may be due to the physical form and dosage of ginger. In their study, diets were supplemented with 10 g/kg ginger powder, which contained not only bioactive compounds but also other nutrients such as carbohydrates and protein [27].

Dietary GE increased albumin height and Haugh unit at both four and eight weeks, which was consistent with the results of Damaziak et al. [28], suggesting that GE might improve albumin quality. This might be attributed to the antioxidant property of gingerols in GE, which probably minimized albumen quality deterioration through lower lipid and protein oxidation [28]. However, previous research indicated that ginger root powder did not affect albumin height or Haugh unit in laying hens at 32 weeks of age [29]. The discrepancy might be due to the difference in laying hen age, which is the most important factor affecting the albumen quality of the freshly laid egg [30]. The effects of ginger might be more obvious for older hens, when albumen quality usually begins to deteriorate. There was no difference in other egg quality traits, indicating that GE did not affect yolk pigmentation, eggshell quality, or egg composition.

There was no difference in fat content of yolk at either four or eight weeks, suggesting that GE did not affect fat deposition in yolk. However, GE decreased yolk cholesterol content only at eight weeks, implying that a period of time might be needed to show the hypocholesterolemic effect of GE. Our data were in accordance with the results of Gurbuz and Salih [17], who reported that ginger root powder reduced yolk cholesterol but not fat content at eight weeks. The hypocholesterolemic effect of ginger was also observed in broilers [31], rats [32], and mice [33]. This might be due to the inhibitory effect of phenolic compounds in GE on 3-hydroxy-3-methylglutaryl coenzyme A, which plays an important role in cholesterol synthesis [16]. In addition, the decrease of yolk cholesterol content may also be attributed to the changes of high-density lipoprotein cholesterol (HDL-C) metabolism, which is involved in reverse cholesterol transport. It was reported that GE could increase serum HDL-C level in rats fed a high-fat diet [34]. Our finding implies that GE may have potential as a feed additive for producing low-cholesterol eggs, which would be preferred by customers because cholesterol is a risk marker for cardiovascular diseases, such as coronary heart disease and stroke [35,36].

Activities of ALT and AST in serum are often used as indicators of liver health because the two enzymes are synthesized in liver and can be released into blood when liver injury occurs [37,38]. In this study, GE decreased activities of ALT and AST in serum, indicating that GE improved liver health of laying hens. Similar results were reported by Malekizadeh et al. [39], who found that 3% ginger rhizome powder supplementation decreased serum ALT and AST activities of laying hens. Such effects of ginger were also observed in broilers [40] and laying quails [41]. The improved hepatic function may be attributed to the antioxidant compounds such as 6-gingerol in GE. Previous studies showed the protective effects of 6-gingerol against liver dysfunction induced by oxidative stress in vitro and in vivo [12,13,42].

The laying hens fed GE had higher T-SOD activity and lower MDA content in yolk at four and eight weeks and in serum, suggesting that GE was effective in improving antioxidant status of laying hens and eggs, which might contribute to increased albumin height and Haugh unit as shown above. Our finding was consistent with the results of Zhao et al. [24], who observed increased T-SOD activity and decreased MDA concentration in yolk and serum of laying hens fed diets supplemented with ginger powder. Yang et al. [26] also reported that increased Haugh unit of eggs was accompanied by improved serum antioxidant status in laying hens fed ginger root. This might be explained by the radical-scavenging activity of antioxidant compounds in GE, which inhibit lipid peroxidation [43] and improve organ function as partly reflected by decreased ALT and AST activities in serum, thus enhancing the synthesis of antioxidant enzymes [14].

## 5. Conclusions

This study confirmed that dietary GE supplementation increased egg weight, and improved albumin height and Haugh unit of eggs, but reduced yolk cholesterol content and activities of ALT and AST in serum at eight weeks. Moreover, the antioxidant status of yolk and serum was also improved by GE. Therefore, GE may be used for the production of eggs with better quality and lower cholesterol level, which is beneficial in decreasing the risk of cardiovascular diseases in humans.

## Figures and Tables

**Table 1:** Ingredient and nutrient composition of the basal diet (g/kg unless otherwise stated).

Items	Content
Ingredient	
Corn	620
Soybean meal	250
Limestone	100
Dicalcium phosphate	12
Methionine	1
Sodium chloride	3
Premix ^1^	14
Nutrient composition	
Metabolizable energy (MJ/kg)	10.81
Crude protein	157
Lysine	7.6
Methionine	3.6
Methionine + cystine	5.4
Calcium	41.7
Available phosphorus	3.1

^1^ Premix supplied per kilogram of diet: transretinyl acetate, 11,000 IU; cholecalciferol, 3500 IU; all-rac-α-tocopherol acetate, 20 mg; menadione, 1.5 mg; thiamin, 1 mg; riboflavin, 6 mg; nicotinamide, 40 mg; choline chloride, 350 mg; calcium pantothenate, 10 mg; pyridoxine·HCl, 2 mg; biotin, 0.04 mg; folic acid, 1 mg; cobalamin, 0.012 mg; Fe (ferrous sulfate), 60 mg; Cu (copper sulfate), 5 mg; Mn (manganese sulfate), 100 mg; Zn (zinc oxide), 65 mg; I (calcium iodate), 0.8 mg; Se (sodium selenite), 0.3 mg.

**Table 2:** Effects of ginger extract on performance of laying hens.

Item	Control	Ginger Extract	SEM ^1^	*p*-Value
Laying rate (%)	87.61	88.95	0.81	0.433
Egg weight (g)	60.14	61.30	0.28	0.031
Egg mass (g)	52.69	54.53	0.55	0.094
Average daily feed intake (g)	111.98	112.69	0.19	0.053
Feed-to-egg ratio	2.13	2.07	0.02	0.185

^1^ SEM, standard errors of means (*n* = 6). Data were analyzed by one-way ANOVA.

**Table 3:** Effects of ginger extract on egg quality of laying hens.

Item	Control	Ginger Extract	SEM ^1^	*p*-Value
4 weeks				
Eggshell strength (kg)	3.06	3.49	0.19	0.270
Eggshell thickness (μm)	286.50	297.44	6.81	0.448
Yolk color	7.52	7.65	0.12	0.592
Albumin height (mm)	6.33	7.70	0.35	0.044
Haugh unit	76.88	85.37	2.20	0.048
Eggshell ratio (%)	12.27	13.08	0.31	0.195
Yolk ratio (%)	25.42	26.65	0.73	0.424
Albumin ratio (%)	62.31	60.26	0.93	0.289
8 weeks				
Eggshell strength (kg)	4.00	3.90	0.16	0.756
Eggshell thickness (μm)	344.56	348.89	7.51	0.788
Yolk color	7.28	7.18	0.16	0.764
Albumin height (mm)	6.32	7.08	0.18	0.024
Haugh unit	80.20	84.53	1.06	0.032
Eggshell ratio (%)	13.95	13.92	0.25	0.954
Yolk ratio (%)	27.24	26.05	0.51	0.262
Albumin ratio (%)	58.81	60.03	0.51	0.256

^1^ SEM, standard errors of means (*n* = 6). Data were analyzed by one-way ANOVA.

**Table 4:** Effects of ginger extract on yolk fat and cholesterol contents of laying hens.

Item	Control	Ginger Extract	SEM ^1^	*p*-Value
4 weeks				
Fat (%)	29.85	28.89	0.50	0.367
Cholesterol (mg/g)	18.66	18.55	0.21	0.810
8 weeks				
Fat (%)	31.79	32.41	0.49	0.553
Cholesterol (mg/g)	20.60	19.27	0.33	0.033

^1^ SEM, standard errors of means (*n* = 6). Data were analyzed by one-way ANOVA.

**Table 5:** Effects of ginger extract on serum transaminase activities of laying hens.

Item ^1^	Control	Ginger Extract	SEM ^2^	*p*-Value
ALT (U/L)	1.81	1.36	0.11	0.023
AST (U/L)	17.60	14.83	0.52	0.002

^1^ ALT, alanine transaminase; AST, aspartate transaminase. ^2^ SEM, standard errors of means (*n* = 6). Data were analyzed by one-way ANOVA.

**Table 6:** Effects of ginger extract on antioxidant status of laying hens.

Item ^1^	Control	Ginger Extract	SEM ^2^	*p*-Value
T-SOD				
4-week yolk (U/g)	93.52	121.02	6.70	0.032
8-week yolk (U/g)	90.79	113.40	4.63	0.006
Serum (U/mL)	296.45	366.24	11.53	<0.001
MDA				
4-week yolk (U/g)	171.66	161.75	2.54	0.045
8-week yolk (U/g)	169.16	149.95	4.33	0.017
Serum (nmol/mL)	6.51	5.11	0.32	0.018

^1^ T-SOD, total superoxide dismutase; MDA, malondialdehyde. ^2^ SEM, standard errors of means (*n* = 6). Data were analyzed by one-way ANOVA.
